# Allopurinol treatment adversely impacts left ventricular mass regression in patients with well-controlled hypertension

**DOI:** 10.1097/HJH.0000000000002189

**Published:** 2019-07-01

**Authors:** Christopher R. Gingles, Ruth Symon, Stephen J. Gandy, Allan D. Struthers, Graeme Houston, Thomas M. MacDonald, Chim C. Lang, Peter T. Donnan, Jacob George

**Affiliations:** a Division of Molecular and Clinical Medicine, University of Dundee; b Department of Clinical Radiology; c Population Health Sciences Division; d MEMO and Hypertension Research Centre, University of Dundee, Dundee, UK

**Keywords:** hypertension, oxidative stress, uric acid

## Abstract

Previous studies have demonstrated that high-dose allopurinol is able to regress left ventricular (LV) mass in cohorts with established cardiovascular disease. The aim of this study was to assess whether treatment with high-dose allopurinol would regress LV mass in a cohort with essential hypertension, LV hypertrophy and well-controlled blood pressure but without established cardiovascular disease.

**Methods:**

We conducted a mechanistic proof-of-concept randomized, placebo-controlled, double-blind trial of allopurinol (600 mg/day) versus placebo on LV mass regression. Duration of treatment was 12 months. LV mass regression was assessed by Cardiac Magnetic Resonance. Secondary outcomes were changes in endothelial function (flow-mediated dilatation), arterial stiffness (pulse wave velocity) and biomarkers of oxidative stress.

**Results:**

Seventy-two patients were randomized into the trial. Mean baseline urate was 362.2 ± 96.7 μmol/l. Despite good blood pressure control, LV mass regression was significantly reduced in the allopurinol cohort compared with placebo (LV mass −0.37 ± 6.08 versus −3.75 ± 3.89 g; *P* = 0.012). Oxidative stress markers (thiobarbituric acid reactive substances) were significantly higher in the allopurinol group versus placebo (0.26 ± 0.85 versus −0.34 ± 0.83 μmol/l; *P* = 0.007). Other markers of vascular function were not significantly different between the two groups.

**Conclusion:**

Treatment with high-dose allopurinol in normouricemic controlled hypertensive patients and LV hypertrophy is detrimental. It results in reduced LV mass regression and increased oxidative stress over a 12-month period. This may be because of an adverse impact on redox balance. Cohort selection for future cardiovascular trials with allopurinol is crucial.

## INTRODUCTION

Essential hypertension is an established risk factor for cardiovascular disease. Furthermore, left ventricular hypertrophy (LVH), which is highly prevalent in treated hypertensive patients [1] is an independent risk factor for cardiovascular events, cardiovascular death and overall mortality [2]. Studies have previously demonstrated that high-dose allopurinol, a xanthine oxidase inhibitor, improves vascular function (and therefore, cardiac afterload) independently of urate [3]. Allopurinol has also been shown to regress LVH in patients with ischemic heart disease [4], chronic kidney disease (CKD) [5] and type 2 diabetes [6]. The mechanism for this LVH regression could either be that allopurinol significantly reduces vascular oxidative stress [3] and improves endothelial function resulting in a reduced cardiac afterload, independent of blood pressure or that allopurinol regresses LVH because of a reduction in oxidative stress, which is a known trigger of myocardial hypertrophy [7,8].

Thus, the main aim of this study was to assess whether high-dose allopurinol could regress left ventricular mass (LVM) in a cohort with essential hypertension and LVH but well-treated blood pressure. The secondary aim was to assess the effect of allopurinol on LV volumes, markers of circulating oxidative stress, endothelial function and arterial stiffness in this patient group.

## METHODS

### Study overview

The study was a single-centre, randomized, double-blind, placebo-controlled mechanistic proof of concept trial with 12 months’ follow-up between 2014 and 2017. The study treatment was allopurinol 600 mg/day, given as 300 mg twice per day or twice daily placebo. The primary objective of the study was to assess LV mass regression after 12 months using cardiac magnetic resonance (CMR). It was approved by the Tayside Research Ethics Committee and was carried out in accordance with the declaration of Helsinki. EudraCT: 2014-002083-33. ClinicalTrials.gov: NCT02237339.

### Study participants

Patients with essential hypertension and LVH were identified from the Scottish Primary Care Research Network, Scottish Health Research Register, Cardiovascular Risk clinics or Cardiology databases. All participants provided informed consent prior to inclusion into the study. Consented participants attended for screening echocardiography to assess for LVH at baseline. LVH was defined by the American Society of Echocardiography criteria as a LVM index (LVMI) greater than 115 g/m^2^ for men and more than 95 g/m^2^ for women [9]. Participants were excluded if they had a documented intolerance to allopurinol, severe aortic stenosis, active gout or taking allopurinol, secondary cause for their hypertension, severe hepatic disease, CKD 3b or worse, patients taking azathioprine, 6 mercaptopurine, or theophylline, active malignancy or other life-threatening diseases, pregnant or lactating women and any contraindication to MRI. Patient were also required to have a baseline daytime average SBP less than 135 mmHg or 24 h average SBP 130 mmHg or less and on stable antihypertensive medications for at least the preceding 3 months prior to randomization.

Eligible participants were stratified for sex and baseline LVMI (men 116–129 g/m^2^ or ≥130 g/m^2^, females 96–114 g/m^2^ or ≥115 g/m^2^) and then randomized using a centrally controlled web-based GCP compliant randomization system (TrusT, Health Informatics Centre, University of Dundee) to receive allopurinol or placebo. Patients continued all other medications including antihypertensive medications.

### Study visits and drug titration

**FIGURE 1:**
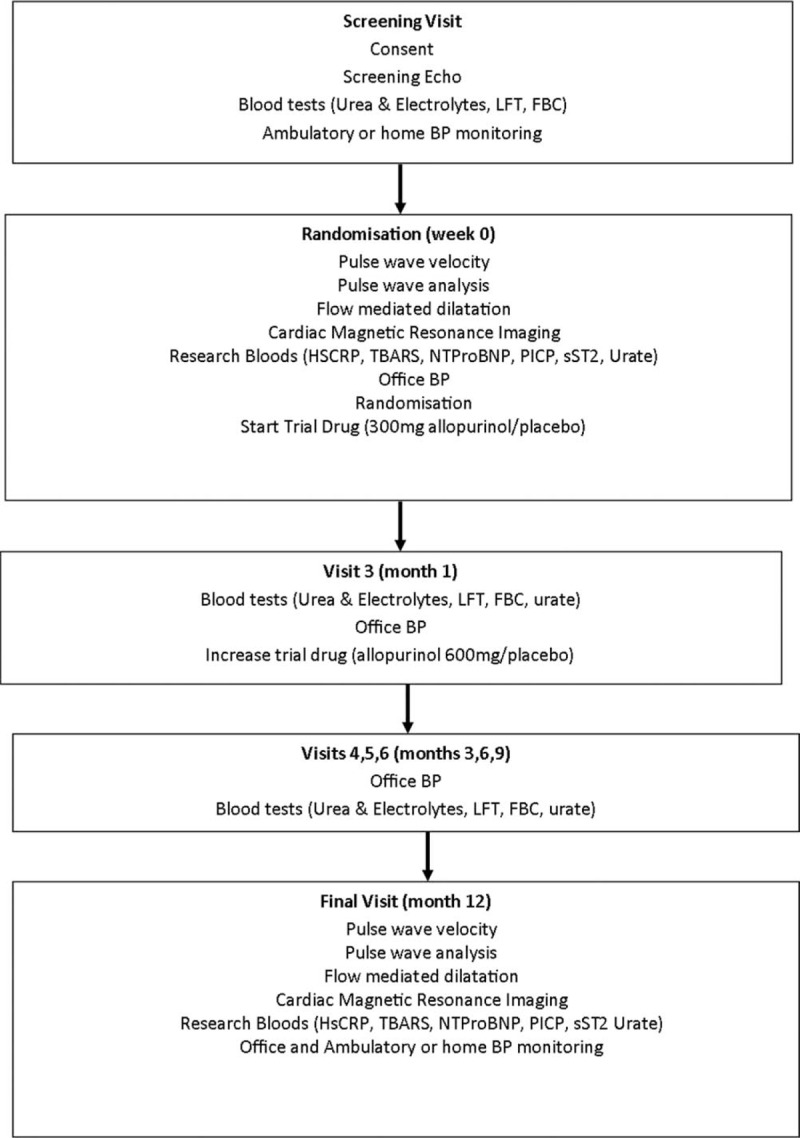
Study visits.

After recruitment, patients attended six further visits over a 12-month period. An initial dosage of allopurinol, 300 mg/day was dispensed, increased to 600 mg/day after 1 month, and continued for the duration of the trial. Study visits are outlined in Fig. 1. Office BP was measured for all patients at each visit, 24-h ambulatory or home BP monitoring was completed in all patients at the start and end of the study.

### Echocardiography

Echocardiographic measurements were made as per the ASE recommendations [10]. Perpendicular linear measurements of the intraventricular septum, internal and posterior wall of the LV were acquired in the parasternal long axis, at the level of the mitral valve leaflets tips at end-diastole. LV mass was calculated using the cube formula.

### Cardiac MRI

Cardiac MRI was performed on a 3T MAGENTOM Trio-Prisma^FIT^ (Siemens, Erlangen, Germany) using dedicated phased array cardiac coils. Serial contiguous short-axis cine images [electrocardiogram-gated (true fast imaging with steady-state precession; TrueFISP)] were acquired from the atrio-ventricular ring to the apex using the vertical and horizontal long axis of the left ventricle as a guide. The short-axis imaging parameters were repetition time (*T*_R_) of 2.5 ms, echo time (*T*_E_) of 1.1 ms, flip angle (FA) of 60°, and slice thickness 6 and 4 mm gap.

Analysis was performed offline by trained observers (C.R.G. and S.J.G.) blinded to the study allocation using Argus software (Version VB15, Siemens Erlangen, Germany). Using the short-axis stack ‘region-of-interest’ contours were placed around the left ventricular endocardial and epicardial borders at end diastole and at end systole to calculate left ventricle ejection fraction (LVEF), left ventricular mass (LVM), end-diastolic (LVEDV), end-systolic (LVESV) and stroke volumes (LVSV). The base and apex were labeled and frames with at least 50% full thickness myocardium were included in the LVM. Papillary muscles were also included in the LVM if the muscle was contiguous with the myocardial wall. Each scan was analyzed twice to ensure consistency, a third measurement was conducted if the LVM varied by more than 5%.

### Flow-mediated dilation

Endothelial function was assessed by measuring flow-mediated dilation (FMD) of the brachial artery using a Sequoia 512 (Siemens, Camberley, UK) and an 8-MHz linear array ultrasound probe. Measurements were conducted at baseline and 12 months according to the International Brachial Artery Reactivity Task Force guidelines [11] by a single operator (C.R.G.) blinded to study allocation.

### Applanation tonometry

Pulse wave velocity (PWV) and augmentation index (AIx) were measured at baseline and 12 months by a single investigator (C.R.G.) blinded to study allocation. Measurements were recorded with a SphygmoCor (AtCor, Sydney, Australia) machine using a high fidelity micromanometer.

### Biomarkers

Blood was collected at the baseline and final visit and analysed for uric acid (colorimetric method; Siemens Healthcare Diagnostics, Erlanger, Germany), N-terminal pro B natriuretic peptide (NTProBNP; multi array assay system; Meso Scale Diagnostics, Maryland, USA), high sensitivity C-reactive protein (HsCRP; ELISA assay, KALON, Aldershot, UK), N-terminal pro b-type natriuretic peptide (PICP; ELISA, Caltag Medsystems, Buckingham, UK), thiobarbituric acid reactive substances (TBARS; trichloroacetic acid method, Cambridge Bioscience, Cambridge, UK) and sST2 (ELISA assay, R&D systems, Minneapolis, Minnesota, USA).

### Statistical analysis

Using data from previous LVH regression studies conducted at our unit [4,6] powered for a similar absolute change in LVM, 58 participants (29 per arm) were required to provide 80% power to detect a 5.2 g difference in LVM between study arms. Data for continuous variable are expressed in means and SDs, and percentages and denominators for categorical variables. Comparison between continuous variables was analysed by the Student *t* test or Mann–Whitney *U* test, categorical variables were analysed by the chi-squared test. Comparison between arms of the trial was assessed by the regression coefficient for the treatment arm with the final visit LVM as the dependent variable and baseline LVM, baseline BP and sex as covariates in a general linear regression model. All statistical analysis was undertaken using SPSS software version 22 (SPSS, Inc., Chicago, Illinois, USA). A *P* value less than 0.05 was considered statistically significant.

### Endpoints

The primary end-point was to assess whether allopurinol regressed left ventricular mass (LVM) in hypertensive patients with controlled blood pressure. Secondary end-points assessed a change in other LV MRI parameters [EDV, ESV, systolic volume (SV), ejection fraction (EF)], parameters of endothelial function and vascular stiffness (FMD, PWV, PWA), biomarkers (uric acid, HsCRP, TBARS, NTProBNP, PICP, soluble ST2), left atrial MRI parameters (EDV, ESV, SV, EF) and BP.

## RESULTS

**FIGURE 2:**
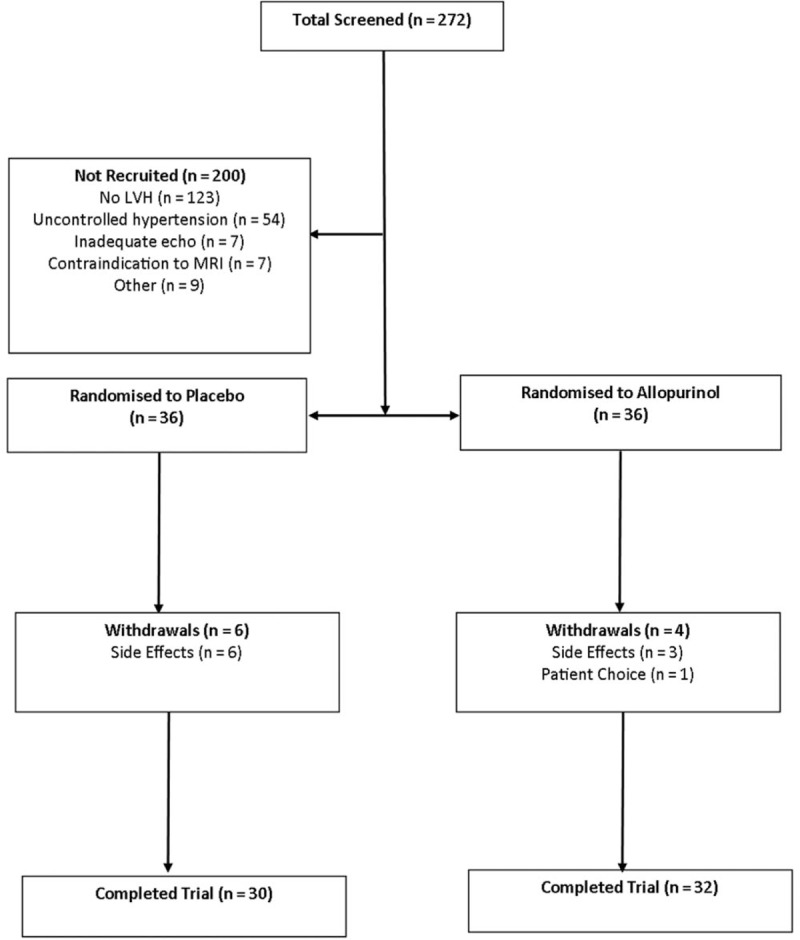
Consort diagram.

In total, 72 participants were recruited (consort diagram; Fig. 2), baseline characteristics for participants who completed the study, including class of antihypertensive treatments are shown in Table 1, with no statistically significant differences between the groups at baseline. Blood pressure control was also well matched at the start and for the whole duration of the trial.

**TABLE 1:** Baseline characteristics of trial participants

Variable	All patients	Placebo	Allopurinol	*P* value
Total patients	*n* = 62	*n* = 30	*n* = 32	
Mean age (years)	65.9 ± 9.4	65.4 ± 9.0	66.4 ± 9.9	0.561
Male	38 (61)	18 (60)	20 (63)	0.840
BMI (kg/m^2^)	30.9 ± 5.1	31.1 ± 5.3	30.7 ± 5.0	0.754
Daytime SBP (mmHg) ABPM/home monitoring	124.8 ± 8.3	125.3 ± 7.5	124.3 ± 9.0	0.620
Daytime DBP (mmHg) ABPM/home monitoring	73.5 ± 8.7	74.1 ± 7.2	73.0 ± 10.0	0.624
Duration of HTN (years)	12.7 ± 8.8	13.4 ± 10.0	12.0 ± 7.6	0.553
IHD	2 (3)	0 (0)	2 (9)	0.164
Dyslipidaemia	27 (44)	14 (47)	13 (41)	0.632
CVA/TIA	7 (11)	4 (13)	3 (9)	0.623
DM	4 (6)	3 (10)	1 (3)	0.271
PVD	1 (1)	1 (3)	0 (0)	0.298
Smoker	4 (6)	3	1	0.521
Ex-smoker	27 (44)	12	15	
Never smoked	31 (50)	15	16	
ACE-I	29 (47)	16 (53)	13 (41)	0.316
ARB	24 (39)	10 (33)	14 (44)	0.400
B blocker	18 (29)	6 (20)	12 (38)	0.129
CCB	44 (71)	22 (73)	22 (69)	0.691
α blocker	14 (23)	6 (20)	8 (25)	0.638
Thiazide diuretic	23 (37)	13 (43)	10 (31)	0.325
Loop diuretic	5 (8)	3 (10)	2 (6)	0.588
MRA	4 (7)	2 (7)	2 (6)	0.947
Centrally acting anti-hypertensive	1 (2)	0 (0)	1 (3)	0.329
Renin blocker	1 (2)	1 (3)	0 (0)	0.298
Number of antihypertensive medications	2.6 ± 1.2	2.6 ± 1.2	2.6 ± 1.3	0.979
Resistant hypertension	14 (23)	6 (20)	8 (25)	0.638
Haemoglobin (g/l)	140.8 ± 12.8	140.2 ± 12.0	141.3 ± 13.7	0.736
Creatinine (mmol/l)	71.2 ± 13.5	73.0 ± 10.9	69.6 ± 15.5	0.315
Glucose (mmol/l)	5.6 ± 0.9	5.4 ± 0.8	5.8 ± 1.0	0.70
Urate (μmol/l)	362.2 ± 96.7	367.3 ± 81.5	357.3 ± 110.1	0.690
HsCRP (mg/l)	2.4 ± 3.3	2.6 ± 3.7	2.3 ± 3.0	0.770
TBARs (μmol/l)	2.8 ± 0.9	2.9 ± 1.0	2.7 ± 0.8	0.342
NTproBNP (ρg/ml)	792.0 ± 891.5	617.8 ± 583.3	960.5 ± 1095.9	0.133
PICP (ng/l)	1.6 ± 0.9	1.7 ± 1.0	1.5 ± 0.7	0.269
Soluble ST2 (ng/ml)	19.9 ± 9.9	19.7 ± 8.1	20.2 ± 11.6	0.834
Echo LVMI (g/m^2^)	125.9 ± 18.7	127.1 ± 21.0	124.8 ± 16.5	0.625
MRI LVM (g)	131.3 ± 36.7	132.5 ± 35.2	130.3 ± 38.5	0.812
MRI LVM Height^1.7^ (g/m^1.7^)	53.2 ± 11.8	54.3 ± 11.9	52.2 ± 11.7	0.489
MRI EDV (ml)	143.5 ± 34.4	142.5 ± 38.0	144.6 ± 31.2	0.815
MRI ESV (ml)	36.9 ± 16.1	36.6 ± 18.9	37.3 ± 13.3	0.862
MRI SV (ml)	106.6 ± 21.6	105.9 ± 22.6	107.3 ± 20.9	0.807
MRI ejection Fraction (%)	75.1 ± 6.1	75.5 ± 7.2	74.7 ± 4.9	0.604
FMD (%)	5.4 ± 3.5	4.9 ± 3.2	5.8 ± 3.8	0.330
AIx (%)	22.3 ± 13.5	21.2 ± 12.7	23.3 ± 14.4	0.534
PWV (m/s)	8.4 ± 1.2	8.2 ± 1.1	8.5 ± 1.4	0.359

Values are *n*, mean ± SD, or *n* (%). ABPM, ambulatory blood pressure monitoring; ACEI, angiotensin converting enzyme inhibitor; AIx, augmentation index; ARB, angiotensin receptor blocker; CCB, calcium channel blocker; CVA, cerebrovascular accident; DM, diabetes mellitus; EDV, end-diastolic volume; ESV, end-systolic volume; FMD, flow-mediated dilation; HsCRP, high-sensitivity C-reactive protein; IHD, ischaemic heart disease; LVM, left ventricular mass; MRA, magnetic resonance angiogram; NTproBNP, N-terminal pro B natriuretic peptide; PICP, N-terminal pro b-type natriuretic peptide; PVD, peripheral vascular disease; PWV, pulse wave velocity; SV, stroke volume; TBARs, thiobarbituric acid reactive substances; TIA, transient ischemic attack.

### Primary endpoint

**FIGURE 3:**
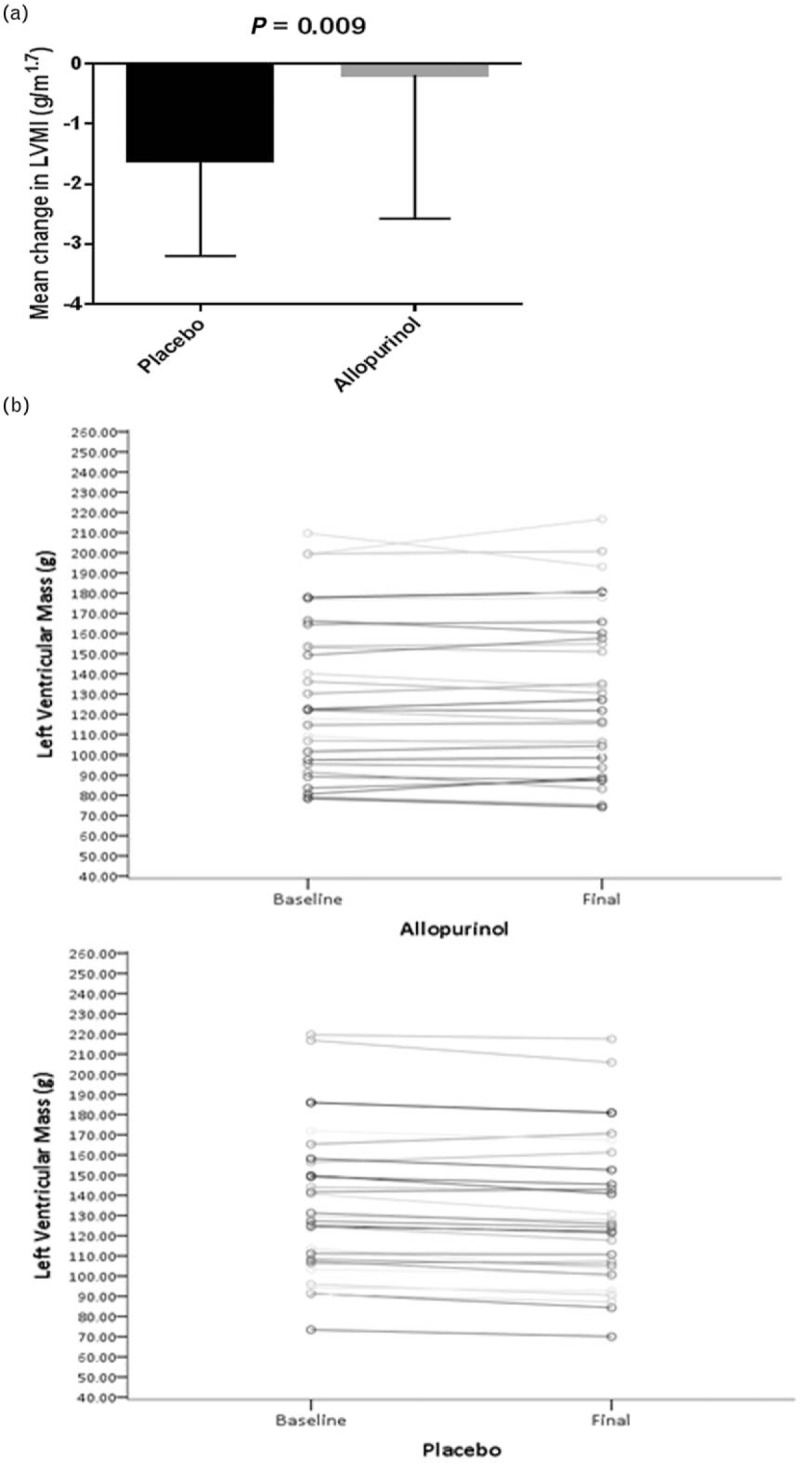
(a) The effect of allopurinol on left ventricular mass index (height^1.7^). Data expressed as mean ± SD. (b) The effect of allopurinol on left ventricular mass.

We found that treating patients with controlled essential hypertension and LVH with allopurinol resulted in potential harm because of significantly reduced LVM regression compared with placebo, individual changes in LVM are illustrated in Fig. 3a and b. The cohort taking allopurinol were found to have a significantly higher final absolute LVM than those taking placebo, after correction for sex, baseline LVM and baseline SBP (Table 2). Forty-five percent of the participants were women. When sex differences were analysed, women on allopurinol had significantly reduced LVM regression compared with men. We did not find a difference between those with high baseline urate versus those with low baseline urate.

**TABLE 2:** Multiple regression: adjusted for sex, baseline SBP, baseline left ventricular mass

Dependent variable	*B* (difference in change)	95% confidence interval		*P*	*R*^2^
		Lower bound	Upper bound		
Absolute LVM (g)	3.43	0.91	5.95	0.008	0.983

LVM, left ventricular mass.

For other parameters measured on cardiac MRI, there were no significant differences in LV ejection fraction, end-diastolic volume, end-systolic volume, stroke volume or left atrial volumes. Sub-group analysis by baseline LV mass, baseline oxidative stress (TBARs) or BP changes all yielded consistent results with the primary analysis.

### Secondary endpoints

There was no statistically significant difference seen in FMD, PWV and PWA between the two treatment arms (Table 3).

**TABLE 3:** Effect of allopurinol on haemodynamics, endothelial function and vascular stiffness

Change in measured parameter	Placebo	Allopurinol	*P* value
24 h systolic (mmHg)	1.2 ± 8.0	0.61 ± 8.0	0.799
24 h diastolic (mmHg)	−0.04 ± 5.40	0.67 ± 4.76	0.634
Daytime systolic (mmHg)	1.57 ± 7.30	−0.94 ± 8.05	0.205
Daytime diastolic (mmHg)	0.07 ± 5.41	0.34 ± 5.72	0.846
FMD (%)	−0.23 ± 3.65	0.14 ± 4.12	0.718
AIx	−0.30 ± 13.46	0.06 ± 12.41	0.913
PWV (m/s)	−0.09 ± 1.12	−0.25 ± 1.07	0.581

AIx, augmentation index; FMD, flow-mediated dilatation; PWV, pulse wave velocity.

### Blood pressure control

Baseline blood pressure, number of antihypertensives (2.5 in placebo arm versus 2.7 in allopurinol arm) and prescribed major classes of antihypertensives was similar between both groups at baseline and remained so throughout the trial. Both cohorts had consistently controlled blood pressure at baseline (125.6 ± 7.4 mmHg placebo versus 124.3 ± 8.8 mmHg allopurinol) and at the end of the trial period (Table 3).

### Biomarkers

The expected significant reduction in uric acid was demonstrated in the allopurinol group (Table 4). There was a significant increase in thiobarbituric acid reactive substances (TBARs), a biomarker for oxidative stress and a nonsignificant increase in high-sensitivity CRP, a marker of inflammation in the allopurinol cohort. There was no significant change between the cohorts for NTProBNP, PICP and soluble ST2 levels (Table 4).

**TABLE 4:** Effect of allopurinol on biomarkers

Change in measured parameter	Placebo	Allopurinol	*P* value
Uric acid (umol/l)	−1.33 ± 37.04	−189.56 ± 91.95	<0.001
HsCRP (mg/l)	−0.55 ± 2.10	0.22 ± 1.71	0.122
TBARS (μmol/l)	−0.34 ± 0.83	0.26 ± 0.85	0.007
NTProBNP (pg/ml)	109.08 ± 491.03	−109.03 ± 612.84	0.131
PICP (ng/l)	−0.18 ± 0.60	−0.05 ± 0.43	0.322
Soluble ST2 (ng/ml)	−1.02 ± 3.39	−0.61 ± 8.63	0.573

HsCRP, high-sensitivity C-reactive protein; NTproBNP, N-terminal pro B natriuretic peptide; PICP, N-terminal pro b-type natriuretic peptide; TBARs, thiobarbituric acid reactive substances.

### Adverse events

There were no suspected unexpected serious adverse reactions (SUSARs). Overall, there were three serious adverse events that required hospital admission; however, all were unrelated to the study medication. Of the three participants who withdrew because of side effects in the allopurinol arm, two developed nausea and one had a rash.

## DISCUSSION

The main finding from this study is that over a 12-month period, allopurinol treatment adversely impacts on the LV mass regression expected with good blood pressure control in patients with hypertension and LVH. Indeed, the expected LVM regression with time because of good blood pressure control was actually reduced in the allopurinol cohort compared with the placebo cohort. We also found that, unlike cohorts with preexisting cardiovascular disease, CKD or diabetes, endothelial function and vascular stiffness did not improve with high-dose allopurinol in this cohort.

Allopurinol has been shown to regress LVM in different cohorts along the cardiovascular spectrum [4–6] with significant preexisting disease, oxidative stress and inflammation. However, the present study has identified that LVM regression may not be seen universally in all populations and that cohort selection of hyperuricemic patients is needed for defining populations that benefit from uric acid lowering. The cohort in this present study was normouricemic. There is a possibility that such patients rely on urate, the most abundant naturally occurring aqueous antioxidant for redox balance.

The results of this present study is consistent with the findings of the Oxypurinol Therapy for Congestive Heart Failure (OPT-CHF) trial where those with uric acid levels of less than 565 μmol/l (9.5 mg/dl) showed a trend towards worsening compared with those with uric acid levels greater than 565 μmol/l [12]. The data relating to urate lowering is also consistent with two recently reported trials, the Febuxostat for Cerebral and Cardiorenovascular Events Prevention Study (FREED) study [13], which suggested the presence of a J-shaped curve with regards to uric acid and clinical events [13] and the Cardiovascular safety of Febuxostat or Allopurinol in Patients with Gout (CARES) study [14], which showed increased all-cause and cardiovascular mortality in the Febuxostat cohort who also had significantly higher proportion of patients achieving uric acid levels less than 0.35 mmol/l, although the latter trial did not prove conclusively that the increase in mortality was related to urate-lowering efficacy.

It is widely accepted that LVH regression occurs with time in patients whose blood pressure is well controlled and this has been confirmed in meta-analysis and systematic reviews of the available trial data [15,16] and incorporated into various national and international guidelines [17]. This is consistent with what we observed in the placebo cohort over a period of 12 months, which showed a significantly greater reduction in LV mass compared with the allopurinol cohort. This, despite the fact that blood pressure in both cohorts remained similar throughout the duration of the study suggesting that any improvement in LVM regression that would have happened in the blood pressure-controlled allopurinol cohort over 12 months was negated by the effect of increased burden of oxidative stress because of reduction in the naturally occurring antioxidant, uric acid, in the allopurinol cohort.

Myocardial hypertrophy is known to be triggered by reactive oxygen species (ROS) and oxidative stress [7,8,18,19]. Therefore, an adverse redox imbalance would be expected to result in an attenuation of the positive impact of good blood pressure control on LVM regression. Data from our group and others have previously suggested that the mechanism by which allopurinol improves LVM regression was mediated by xanthine oxidase inhibition and the consequent reduction in oxidative stress in cohorts with background ischemic heart disease, CKD and type-2 diabetes mellitus [4–6]. The LVH regression in these previous cohorts was also associated with an improvement in vascular endothelial function suggesting potential impact on vascular stiffness and cardiac afterload, independent of blood pressure.

The lack of LVM regression in this present cohort, unlike other cohorts we have previously studied, suggests that reducing uric acid in normouricemic patients but without established vascular disease, and therefore low-background oxidative stress or inflammation could tip the anti-oxidant pro-oxidant balance negatively and cause detriment.

This might be an explanation for our findings as uric acid is the most abundant naturally occurring aqueous antioxidant in humans and contributes as much as two-thirds of all free radical scavenging capacity in plasma [20,21]. Uric acid can, however, switch from an antioxidant to pro-oxidant under certain conditions, such as ischemia and inflammation, termed the uric acid paradox [22] or the uric acid redox shuttle [23–25]. Whether lower doses of allopurinol may be less detrimental in this specific cohort requires further investigation. We selected this high dose (600 mg/day) as previous trials using this dose had shown LVM regression in cohorts with established disease [4–6].

We stress that the results of this study is a preliminary indication of a possible mechanism. Further in-depth research is required to evaluate if indeed a redox imbalance directly results in a reduction in LV mass regression. This study suggests that reducing uric acid had a detrimental impact on redox balance and of ROS levels and myocardial structure, consistent with findings of previous trials, such as FREED [13], CARES [14] and OPT-CHF [12] as well as the redox shuttle uric acid paradox previously described [22–25]. This is further suggested, but not definitively confirmed, by our finding that TBARs were significantly increased in the allopurinol cohort. A multiple regression analysis with treatment, BP changes and oxidative stress changes revealed that increasing TBARs impacted final LVM. We found an increased but not statistically significant, level of hs-CRP in the allopurinol cohort suggesting the possibility of downstream inflammatory impact of increased ROS. It is reassuring that when the small number of patients with pre-existing vascular disease was excluded, the results were consistent with the primary analysis for all measures (LV mass index, augmentation index and change in FMD).

### Study limitations

This is a single-centre study. Screening for LVH was done by echocardiogram by a qualified echocardiographer. Due to the cost of CMR, it was not used as a screening tool. However, LV mass regression was determined by cardiac MRI, the current gold standard modality for this purpose. Reassuringly, the baseline CMR-determined LV mass was comparable with previously published studies on LV Mass regression on other cohorts [4–6].

As the results of this study contrast with the beneficial effect of allopurinol in LVH regression seen in patients with IHD, type 2 diabetes and CKD, we acknowledge that there is always a possibility that the results of this study may have occurred by chance. However, the lack of LVM regression is consistent with results in all other CMR, vascular stiffness and endothelial function measures we studied. Furthermore, the worsening of oxidative stress burden seen with the TBAR levels, although a secondary outcome, and therefore not powered, lends support to the findings.

In conclusion, this study, in conjunction with previously published data, including the OPT-CHF trial [12], CARES [14] and FREED [13] suggests that the cardiovascular benefits of allopurinol are mainly seen in hyperuricemic, high oxidative stress states driven by pre-existing ischemia and/or inflammation. Therefore, cohorts for future large cardiovascular outcomes trials with allopurinol should be carefully selected based on only populations that have demonstrated benefit.

## ACKNOWLEDGEMENTS

Author contributions: A.D.S. and J.G. conceived the idea for the study. A.D.S., J.G., S.J.G., T.M.M., C.C.L. and J.G.H. designed the study. C.R.G., R.S. and S.J.G. performed research. S.J.G., P.T.D. and C.R.G. analyzed the data. All authors contributed to the writing of the manuscript.

Sources of funding: This study was funded by a grant from the British Heart Foundation (PG/13/67/30444). This trial was supported by the Tayside Clinical Trials Unit.

Clinical Trial Registration: ClinicalTrials.gov Identifier: NCT02237339;

ISRCTN number: ISRCTN40476871.

EUDRACT: https://www.clinicaltrialsregister.eu/. Unique identifier: 2014-002083-33.

### Conflicts of interest

A.D.S. and the University of Dundee have applied for a patent on the use of xanthine oxidase inhibitors to treat angina pectoris. All other authors have no conflicts of interest.
